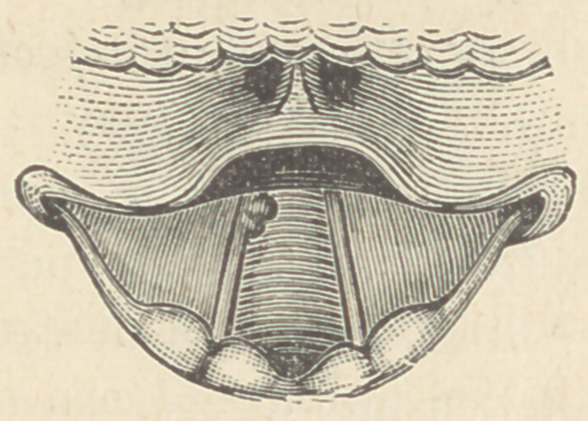# Chicago Medical Society

**Published:** 1879-04

**Authors:** R. Park


### ARTICLE IX.

CHICAGO MEDICAL SOCIETY. Reported by R. Park, M.D. Meeting at the Grand Pacific Hotel, March 3d, 1879. E. Ingals, M.D., in the chair.

The committee appointed at the last meeting to witness the demonstrations by Dr. Ephraim Cutter of specific spores in the blood of phthisical and syphilitic patients made their report. The committee thought that the demonstrations failed to establish Dr. Cutter's theories.

DR. D. W. GRAHAM presented the larynx of a patient who had died in a paroxysm of dyspnœa, with the following history:

Flora L., eight years of age, of English parentage, a decided blonde, of anæmic appearance. She has always had fair general health.

There is evidence of syphilitic taint on the paternal side of the family.

About four years ago I removed a large warty mass from the anus of a younger brother of the patient, which presented all the characteristics of the ordinary venereal wart. With these exceptions, the family history is good.

Three years ago the patient became hoarse, and the power of phonation gradually disappeared, leaving only a faint whisper. There was no dyspnœa at any time, except two paroxysms, of short duration, the one which terminated fatally and one a week previous.

On examining the specimen, we find the mucous membrane of the upper vocal cords, the ventricles, the true vocal cords, and of the inferior cavity as low down as the cricoid cartilage, covered with a warty looking excrescence, best developed, however, on the true vocal cords. The lower left border of the patch is slightly ulcerated and purulent.

After microscopical examination, Dr. I. N. Danforth pronounces the growth papilloma.

This patient has been under my observation at intervals for over a year and a half. The last time I saw her was in November last. I prescribed iodide of potassium and tonics from the first, which were taken very irregularly.

The diagnosis, although suspected, was never made. Repeated efforts and appointments for examination with the laryngoscope were made, but failure of the parents to keep the appointments caused me to lose interest in the case.

With the patient well in hand, and the co-operation of the parents, there is no reason why this case should not have been accurately diagnosticated.

This accomplished, under the same conditions, the case could have been, I think, without doubt successfully treated; and I confess to some degree of self-reproach in the matter for not more fully appreciating the danger of sudden death, and for not more urgently persuading the parents into co-operation.

DR. E. FLETCHER INGALS reported two cases of laryngeal tumors, which he had recently removed.

*Case* 1.—Fibrous tumor on the left vocal cord; partial evulsion; cure.

Miss D. H., æt. 23, seamstress, applied at the Central Free

Dispensary, on account of hoarseness and occasional pain in swallowing. Upon examination with the laryngoscope, I found the larynx considerably congested, the vocal cords swollen and congested, and a small, slightly pedunculated tumor growing from the under surface of the left vocal cord near its anterior extremity. The application of astringent powders soon removed the swelling of the vocal cord, and seemed to cause slight diminution in the size of the tumor. The larynx was very sensitive and intolerant of instruments.

In October, 1877, the hoarseness had existed ten or eleven months; all acute inflammatory symptoms had subsided but the tumor still remained. After frequent attempts to seize the tumor with McKenzie's tube forceps, with blades opening antero-posteriorly, and failure to pass the blades between the vocal cords on account of the nearness of the tumor to the anterior commissure, I determined to seize that portion of the tumor (about two-thirds) which rose above the vocal cords. Turning the blades so that they opened laterally, I seized the tumor, crushing it firmly, and bringing away a portion of its mucous covering. Three days later the tumor had disappeared, but there was a prominence at its original seat, due to congestion and swelling of the cord. Ten days later the cord had resumed its normal appearance, and the voice was completely restored.

*Case* 2.—Fibro-cellular tumor on the right vocal cord, with partial aphonia; evulsion; cure.

Mrs. S., æt., 20. Formerly a teacher and elocutionist. Has been troubled for fifteen month with hoarseness, which seemed to have its origin in a common cold. Complained also of occasional dull pains in the larynx. The patient was treated for several months with gargles, inhalations, etc., and latterly has been treated, by a prominent homœopathic physician of this city, for chronic laryngitis, with electricity, the poles being applied externally over the larynx.

Upon examination, I found a small tumor growing from the under surface of the right vocal cord, and situated about three millimeters from its anterior extremity. The tumor, as represented in the cut measured about six millimeters at its base and three at its apex.

During ordinary respiration the epiglottis hung so far backward that it was impossible to see more than a small portion of the posterior part of the glottis, but while the patient phonated a high pitched *a*, the epiglottis was raised and about half of the tumor projected above the vocal cord. In this case, also, I found it impossible to grasp the tumor with the blade of the forceps opening antero-posteriorly.

The position of the epiglottis rendered it impossible to pass the forceps into the larynx during ordinary respiration, and even during deep respirations the end of the forceps could not be carried far enough forward to reach the tumor without firm pressure by the instrument on the epiglottis, which would produce immediate closure of the larynx.

Accordingly, I was obliged to grasp the tumor during phonation, by which means I succeeded in removing the greater portion of it. The tumor when removed was much reduced in size, its semi-fluid contents having been forced out by the forceps.

Five days after the operation there was no trace of the tumor, though the cord was somewhat congested and swollen. Six days later nearly all congestion had disappeared, and the voice seemed normal, though the patient's mother thought it still a trifle husky. A few days later the patient assured me that the voice was perfect.

Dr. F. H. Davis presented to the society the results of his observation and study in the adpatation of local treatment to pulmonary diseases.

The chief obstacle encountered was in the lack of tolerance of the deeper air passages to local medication. The admixture of any medicament, either in gaseous, liquid or solid form with the inspired air, was immediately resented by the lungs in an involuntary, spasmodic cough, or attempt at rejection of the irritant material.

For this reason the various gases and vapor, or spray treatments, which had been from time to time put forward, had invariably disappointed the high hopes theoretically entertained for them, and had mostly fallen into disuse.

The author believes however, that in a certain limited range of pulmonary diseases a properly adopted local treatment would insure results not obtainable by any other form of treatment.

In acute inflammations of the air passages, moist or steam inhalations were considered to be contra-indicated on account of their tendency to increase the sub-mucous infiltration and œdematous swelling. In chronic, suppurative bronchitis, the inhalation of air charged with steam and carbolic acid vapor would readily check the excessive expectoration,—the addition of an anodyne, as camph. tincture of opium, would further lessen the irritative cough. The same result could, however, be perhaps as certainly and as speedily reached by the ordinary internal treatment.

The class of cases where local inhalations were chiefly of value, were those presenting the symptoms of incipient tuberculosis, or those approaching the second or suppurative stage, and also in cases of pueumonic inflammation tending, on account of the lack of recuperative power in the system, to take on a degenerative suppurative action to end in inflammatory phthisis.

In all of these cases it was of the utmost importance to conserve all the power and strength of the digestive organs for the building up and support of the system by food, nutritive tonics, etc., and the less these organs were taxed with ordinary medicines—anodynes, expectorants and the usual cough mixtures, the better for the patient.

The inhalation of air charged with warm steam and the vapor of paregoric with the addition, in incipient tuberculosis, of some one of the balsamic preparations, as the oil of Scotch pine or the oil of English juniper, was recommended. If suppurative action had commenced, carbolic acid was substituted for the balsam.

The following formulas were given by the author.

| | | | |
|---|---|---|---|
| ℞ | Oil Scotch pine | 5 | 0 |
| | Tr. opii. camph | 100 | 0 |

Sig.—1 teaspoonful to be added to ½ pint of boiling water, and the vapor inhaled for five minutes two or three times a day or oftener.

| | | | |
|---|---|---|---|
| ℞ | Acid. carbolic. cryst | 2 | 0 |
| | Tr. opii. camph | 100 | 0 |

Sig.—Use the same as above

A tin inhaler which was exhibited was claimed to possess the following advantages: Large tubes (¾ inch) which permitted free inhalation without suction; valves so arranged as to enable the patient to expire slowly and against a certain amount of pressure which exerted an expansive effect upon the lungs and held the inhaled vapor longer in contact with the air passages. Lastly and chiefly, cheapness—the inhalers being sold to patients at seventy-five cents each. These inhalers are on sale by D. R. Dyche & Co., State and Randolph Sts., Chicago.

These inhalers on first being used created some reflex irritative cough, but this was quickly subdued by the anodyne effect of the opiate, and combined with the stimulant effect of the balsams or the disinfecting influence of the carbolic acid they would be found to accomplish most gratifying and satisfactory results in a class of cases otherwise unmanageable.

## Figures and Tables

**Figure f1:**
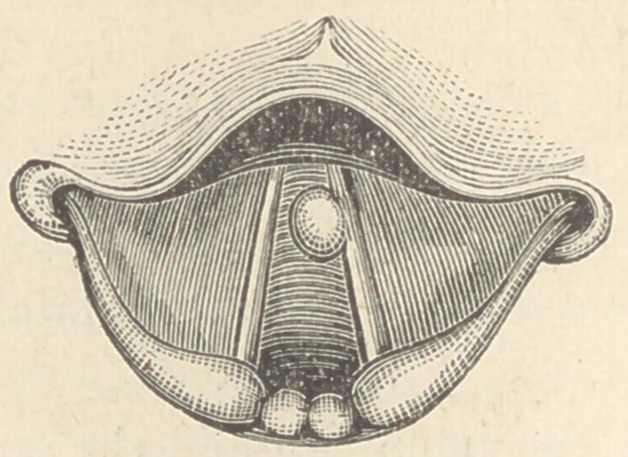


**Figure f2:**